# Low serum amylase in association with metabolic syndrome and diabetes: A community-based study

**DOI:** 10.1186/1475-2840-10-34

**Published:** 2011-04-17

**Authors:** Kei Nakajima, Tohru Nemoto, Toshitaka Muneyuki, Masafumi Kakei, Hiroshi Fuchigami, Hiromi Munakata

**Affiliations:** 1 Division of Clinical Nutrition, Department of Medical Dietetics, Faculty of Pharmaceutical Sciences, Josai University, 1-1 Keyakidai, Sakado, Saitama, 350-0295, Japan; 2 First Department of Comprehensive Medicine, Saitama Medical Center, Jichi Medical University School of Medicine, 1-847 Amanuma, Omiya, Saitama 330-8503, Japan; 3 Department of Health Care Center, Social Insurance Omiya General Hospital, 453 Bonsai, Kita, Saitama, 331-0805, Japan; 4 Department of Internal Medicine, Social Insurance Omiya General Hospital, 453 Bonsai, Kita, Saitama, 331-0805, Japan

**Keywords:** serum amylase, exocrine, impaired insulin action, metabolic syndrome, diabetes, kidney function, GFR

## Abstract

**Background:**

Low serum amylase levels may reflect impaired exocrine-endocrine relationship in the pancreas. However, few clinical studies have addressed this issue. Therefore, in this epidemiological study, we investigated whether low serum amylase was associated with the pathogenesis of impaired insulin action: metabolic syndrome (MetS) and diabetes.

**Research Design and Methods:**

Serum amylase, cardiometabolic risk factors, MetS (Adult Treatment Panel III criteria), and diabetes were examined in 2,425 asymptomatic subjects aged 30-80 years who underwent medical checkups recently (April 2009-March 2010) and 5 years ago.

**Results:**

Clinical variables, except for age and estimated glomerular filtration rate (eGFR), shifted favorably with increasing serum amylase levels. Plasma glucose levels at 1- and 2-hr during OGTT increased significantly with decreasing serum amylase levels. Multiple logistic analyses showed that, compared with highest quartile of serum amylase, lowest quartile was associated with increased risk for MetS and diabetes after adjustment for confounding factors [odds ratio (95% CI), 2.07 (1.39-3.07) and 2.76 (1.49-5.11), respectively]. In subjects who underwent checkups 5 years ago (n = 571), lower amylase at the previous checkup were associated with larger numbers of metabolic abnormalities at the recent checkup. The fluctuation over time in serum amylase levels in subjects with low serum amylase at the previous checkup was slight and was unaffected by kidney dysfunction.

**Conclusions:**

Our results indicate that low serum amylase is associated with increased risk of metabolic abnormalities, MetS and diabetes. These results suggest a pancreatic exocrine-endocrine relationship in certain clinical conditions.

## Introduction

Elevated serum amylase levels often accompany acute pancreatitis [1-3] and are occasionally caused by other conditions such as pancreatic tumors, diabetic ketoacidosis and kidney dysfunction [1-6]. In contrast, low serum amylase is thought to be due to diffuse pancreas destruction secondary to advanced chronic pancreatitis or alcoholic disease [7-9]. Low serum amylase is also associated with insulin deficiency in patients with type 1 diabetes and, less commonly, with type 2 diabetes [10-13], as well as the pathogenesis of insulin resistance in obese animal models [14,15]. Because serum amylase can be classified as pancreatic-type and salivary-type amylase [2,3], it is often necessary to distinguish between the two types for precise clinical diagnosis. Nevertheless, the measurement of serum amylase is useful to determine the pathogenesis of many diseases.

To date, the clinical relevance of low serum amylase levels remains poorly understood. Animal and cellular studies regarding the relationship between the endocrine and the exocrine pancreas have consistently showed that insulin affects basal and stimulatory amylase secretion via the islet-acinar axis [16-19]. Briefly, insulin binds to its receptor on acinar cells and stimulates amylase secretion through various pathways [17-19]. However, the nature of this relationship between low serum amylase and clinical conditions has been addressed by relatively few, small-scale human studies that yielded some conflicting results [10-13,19,20].

To date, no large-scale epidemiological studies have been conducted to explore the relationship between low serum amylase levels and cardiometabolic diseases associated with insulin resistance and/or inadequate insulin secretion, such as metabolic syndrome (MetS) and diabetes. Therefore, the aim of this epidemiological study was to determine whether low serum amylase levels are associated with cardiometabolic risk factors, MetS, and diabetes, which could yield insight into a possible exocrine-endocrine relationship in clinical conditions. In this context, we examined these associations in a cross-sectional community study of asymptomatic adults. In addition, because some proportion of the participants underwent checkups 5 years ago, we retrospectively evaluated whether the previous low serum amylase was associated with MetS, diabetes, and changes in metabolic abnormalities over time. Fluctuations in serum amylase levels were also examined according to kidney function assessed by estimated glomerular filtration rate (eGFR), which often deteriorates following the development of MetS and diabetes, and is a key factor that increases serum amylase [2-6].

## Methods

### Subjects

The subjects in this study were asymptomatic adults aged 30-80 years who lived in the suburbs of Saitama, Japan, and underwent thorough medical checkups at Social Insurance Omiya General Hospital, Saitama, Japan. The present report represents a series of observational studies performed in collaboration with Josai University, Sakado, Japan and Social Insurance Omiya General Hospital that have been conducted using data collected between April 2009 and March 2010 to elucidate the relationships between lifestyle-related diseases and cardiometabolic factors. The protocol was approved by The Ethics Committee of Josai University and the Council of the Hospital, and informed consent was obtained from all participants. All subjects who underwent a medical checkup were recruited in this study, without any special selections. However, subjects with C-reactive protein (CRP) ≥ 10.0 mg/l, eGFR ≤ 35 ml/min/1.73 m^2^, serum amylase ≤ 30 IU/l or ≥ 200 IU/l, and those suspected of having cancer or endocrinopathies were excluded from the study. Consequently, a total of 2,425 individuals were included in this study, which included 571 individuals who underwent checkups 5 years ago.

### Anthropometric and laboratory measurements

Blood tests, anthropometric tests, blood pressure tests and abdominal ultrasound scans (to screen for liver, gallbladder, pancreas and adrenal gland diseases) were carried out after an overnight fast. Serum parameters were measured using an autoanalyzer (Hitachi. Tokyo, Japan). The serum amylase level was measured using an enzymatic method (L-type Amylase, Wako, Tokyo, Japan) with a normal range of 41-112 IU/l, a detection limit of 1.7 IU/l, and a run-to-run coefficient of variation < 5.0%. Plasma glucose was measured by the glucose oxidase method. HbA1c was measured in Japan Diabetes Society (JDS)-HbA1c units by high-performance liquid chromatography. HbA1c was converted to National Glycohemoglobin Standardization Program (NGSP) levels by the formula HbA1c (%) (NGSP) = HbA1c (JDS) (%) + 0.4%, considering the relational expression of HbA1c (JDS) (%) measured by the previous Japanese standard substance and measurement methods [21].

eGFR was calculated using the Modification Diet in Renal Disease study equation for Japanese subjects [22], as follows:

Cr: serum creatinine concentration (mg/dl).

The diagnosis of MetS was based on the Adult Treatment Panel (ATP) III criteria [23] with the following cutoff limits: 1) systolic blood pressure ≥ 130 mmHg or diastolic blood pressure ≥ 85 mmHg; 2) triglyceride (TG) ≥ 150 mg/dl; 3) low high-density lipoprotein cholesterol (HDL-C) < 40 mg/dl for men and < 50 mg/dl for women; 4) fasting plasma glucose (FPG) ≥ 100 mg/dl; and 5) waist circumference ≥ 90 cm for men and ≥ 80 cm for women. Subjects meeting three or more of these criteria were defined as having MetS. If subjects were treated for any of these components, they were determined to meet that criterion. The number of ATP-III-MetS components (NAMC) was recorded as an indicator reflecting the severity of metabolic abnormalities. Diabetes was defined as FPG ≥ 126 mg/dl or HbA1c ≥ 6.5% according to the American Diabetes Association diagnostic criteria [24], or being treated with oral hypoglycemic drugs or insulin. Most of the patients with diabetes in this study were considered to be type 2 diabetes because this is much more prevalent (90-95%) [24], although the exact diagnosis was not confirmed. Subjects with prediabetes, those suspected of having diabetes, and those who wished to know their glucose metabolic status in more detail, underwent a 75-g oral glucose tolerance test (OGTT). Consequently, 1,244 subjects (856 men and 388 women) underwent the test. Of these subjects, 23 subjects were diagnosed as diabetes afterward with a mean HbA1c of 6.4%.

### Retrospective longitudinal subanalysis

A total of 707 subjects actually underwent a checkup at the same hospital 5 years ago, and most of the parameters recorded at the current checkup, except for waist circumference, were recorded previously. However, 136 subjects had already had MetS and/or diabetes, which was retrospectively diagnosed with the identical criteria described above, thereby those subject were excluded from the cohort data. Consequently, 571 subjects remained for the retrospective longitudinal subanalysis. Because waist circumference was not recorded at the previous checkup, body mass index (BMI) ≥ 25 kg/m^2 ^at that time was included in the MetS criteria instead of elevated waist circumference. Accordingly, such NAMC is termed as NAMC-BMI. Changes during the periods in NAMC-BMI and proportions of its components [elevated blood pressure, dyslipidemia (high TG and/or low HDL), and elevated FPG] were examined according to the quartiles of serum amylase. In addition, changes in serum amylase during the periods were also assessed according to the quartiles of serum amylase at the previous checkup and three categories divided by current kidney function.

### Statistical analysis

The data are expressed as means ± SD or median/geometric mean (interquartile range). Multiple stepwise regression analysis was conducted to determine parameters that significantly explain the serum amylase level. Dichotomous parameters were labeled as 0 and 1, respectively, before including as independent variables. Subjects were divided into quartiles of serum amylase for current and previous levels. Clinical variables were compared with one-way analysis of variance (ANOVA). Serum amylase was examined with two-way ANOVA with four categories consisting of NAMC (0, 1, 2, and 3-5) as one factor and smoking status as another factor. OGTT blood glucose levels were similarly examined by two-way repeated ANOVA. In the 571 subjects included in the longitudinal analysis, changes in NAMC-BMI over time were examined by one way and two-way ANOVA. Multivariate logistic regression models were used to examine the associations between lowest amylase quartile with MetS and diabetes after adjustment for confounding factors (current and historical parameters). Statistical analysis was performed using SPSS software version 18.0 (SPSS-IBM Chicago, IL). Values of p < 0.05 were considered statistically significant.

## Results

**Figure 1:**
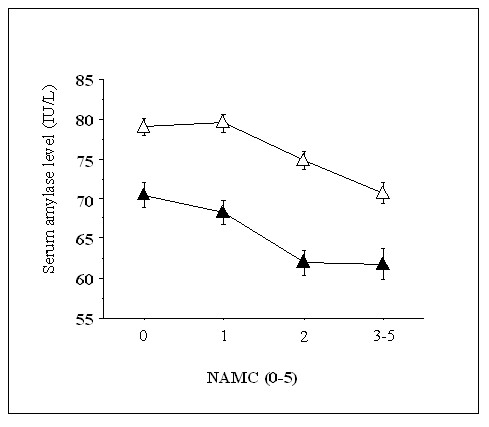
**Serum amylase levels according to the number of ATP-III-MetS components (NAMC)**. The numbers of nonsmokers with NAMCs of 0, 1, 2 and 3-5 are 472, 580, 438 and 306, respectively. The numbers of smokers with NAMCs of 0, 1, 2 and 3-5 are 169, 184, 144 and 132, respectively. The serum amylase level decreased significantly with increasing NAMC in both nonsmokers and smokers (both *P *< 0.0001, ANOVA), and was significantly different between nonsmokers and smokers (*P *< 0.0001, ANOVA). White triangles = nonsmokers, black triangles = smokers. Values are means ± SE.

**Figure 2:**
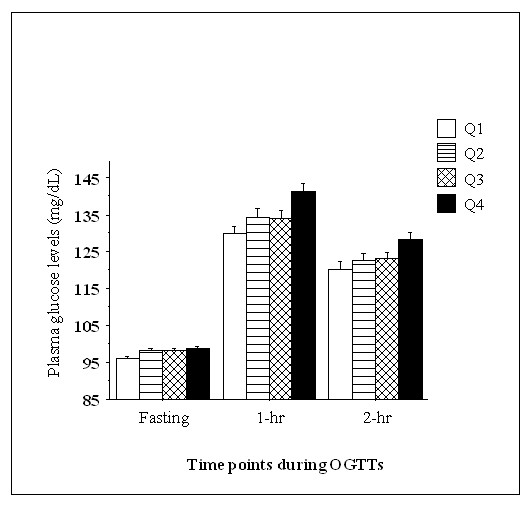
**Plasma glucose levels during OGTTs**. The numbers of subjects in Q1, Q2, Q3 and Q4 are 290, 312, 322 and 320, respectively. The cutoff values for each quartile are presented in Table 1. Plasma glucose levels increased significantly with decreasing serum amylase levels (*P *= 0.002, ANOVA). Values are means ± SE.

The clinical characteristics of subjects according to serum amylase quartile are shown in Table 1. Across increasing amylase quartiles, the prevalence of men, smokers, daily alcohol consumption, infrequent exercise, MetS, and diabetes decreased significantly. Most clinical variables were decreased (HDL-C was increased) across increasing amylase quartiles, except for age and eGFR which showed unfavorable changes. Intriguingly, the prevalence of history of stroke and medications for hypercholesterolemia were higher in the highest quartile. Unexpectedly, the statistical significance for the relationship between HbA1c and serum amylase was weaker (*P *= 0.02) than that of other variables. Of clinical parameters listed in Table 1, stepwise regression analysis revealed that serum amylase level was significantly explained by BMI, eGFR, smoking, age, daily alcohol consumption, FPG, diastolic blood pressure, and triglyceride (ß coefficient = -0.17, -0.17, -0.11, 0.11, -0.07, -0.05, -0.05, and -0.04, respectively, data not shown). Figure 1 shows serum amylase levels according to NAMC categories. In both non-smokers and in smokers, serum amylase levels decreased significantly with increasing NAMC almost in parallel, with an approximately 10 IU/l decline in smokers. Figure 2 shows the changes in plasma glucose levels during OGTTs according to serum amylase quartiles. Because subjects with definite diabetes did not undergo OGTT, overall responses to OGTT showed a near-normal pattern. Nevertheless, the plasma glucose levels at 1- and 2-hrs increased significantly with decreasing amylase quartiles.

**Table 1:** Characteristics of subjects according to serum amylase quartiles

	Total	Q1 (Lowest)	Q2	Q3	Q4 (Highest)	*P *value
n	2,425	610	585	613	617	
Men, n (%)	1,589 (65.5)	428 (70.2)	419 (71.6)	376 (61.3)	366 (59.3)	< 0.0001*
Age, y	53.8 ± 11.9	51.1 ± 11.0	51.9 ± 11.4	54.6 ± 12.1	57.4 ± 12.0	< 0.0001
BMI, kg/m^2^	23.3 ± 3.2	24.3 ± 3.4	23.5 ± 3.1	23.1 ± 3.1	22.3 ± 2.9	< 0.0001
Waist circumference, cm	82.1 ± 8.8	84.6 ± 9.0	82.9 ± 8.5	81.7 ± 8.7	79.2 ± 8.3	< 0.0001
Systolic blood pressure, mmHg	122 ± 19.0	125 ± 19.2	121 ± 18.2	121 ± 19.4	120 ± 18.8	0.0002
Diastolic blood pressure, mmHg	75.7 ± 12.8	78.1 ±13.1	76.0 ± 12.8	74.9 ± 12.4	73.9 ±12.4	< 0.0001
γ-glutamyltransferase, IU/l	26 (18-45)	32 (22-55)	28 (18-48)	24 (17-40)	23 (16-38)	< 0.0001
Amylase, IU/l (range)	74.0 ± 23.6 (31-193)	48.7 ± 6.3 (31-57)	63.4 ± 3.5 (58-69)	77.5 ± 4.8 (70-86)	106 ± 19.1 (87-193)	--
Total cholesterol, mg/dl	205 ± 33.9	205 ± 33.7	204 ± 33.9	205 ± 32.8	208 ± 35.3	0.18
Triglyceride, mg/dl	94 (68-136)	110 (75-159)	95 (69-140)	92 (66-132)	85 (63-123)	< 0.0001
HDL-C, mg/dl	61.0 ± 14.9	58.3 ± 14.2	59.9 ± 14.1	61.7 ± 15.4	63.9 ± 15.3	< 0.0001
Fasting plasma glucose, mg/dl	101 ± 18.1	104 ± 23.0	101 ± 18.3	99.8 ± 14.8	98.8 ± 14.6	< 0.0001
HbA1c, %	5.75 ± 0.61	5.81 ± 0.79	5.75 ± 0.65	5.71 ± 0.45	5.74 ± 0.51	0.02
CRP, mg/l	0.53 (0.30-0.70)	0.62 (0.30-1.15)	0.51 (0.30-0.70)	0.52 (0.30-0.80)	0.47 (0.30-0.70)	< 0.0001
eGFR, ml/min/1.73 m^2^	76.0 ± 13.9	79.7 ± 13.2	77.7 ± 13.5	75.0 ± 13.3	72.0 ± 14.4	< 0.0001
Metabolic syndrome, n (%)	438 (18.1)	155 (25.4)	111 (19.0)	105 (17.1)	67 (10.9)	< 0.0001*
Diabetes, n (%)	201 (8.3)	75 (12.3)	47 (8.0)	38 (6.2)	41 (6.6)	0.0004*
Medical history of						
Cardiovascular diseases, n (%)	85 (3.5)	19 (3.1)	13 (2.2)	26 (4.2)	27 (4.4)	0.14*
Stroke, n (%)	41 (1.7)	6 (1.0)	5 (0.9)	10 (1.6)	20 (3.2)	0.004*
Medications for						
Hypertension, n (%)	413 (17.0)	103 (16.9)	98 (16.8)	96 (15.7)	116 (18.8)	0.52*
Hypercholesterolemia, n (%)	254 (10.5)	61 (10.0)	55 (9.4)	51 (8.3)	87 (14.1)	0.006*
Diabetes, n (%)	81 (3.3)	23 (3.8)	16 (2.7)	18 (2.9)	24 (3.9)	0.59*
Current smoker, n (%)	629 (25.9)	236 (38.7)	177 (30.3)	125 (20.4)	91 (14.7)	< 0.0001*
Daily alcohol consumption, n (%)	757 (31.2)	224 (36.7)	203 (34.7)	170 (27.7)	160 (25.9)	< 0.0001*
Infrequent exercise, n (%)	1,650 (68.0)	448 (73.4)	405 (69.2)	416 (67.9)	381 (61.8)	0.0002*

BMI, body mass index; HDL-C, high-density lipoprotein cholesterol; CRP, C-reactive protein; eGFR, estimated glomerular filtration rate. Data are means ± SD. γ-glutamyltransferase, triglyceride and CRP are expressed as medians/geometric means (interquartile range). γ-glutamyltransferase, triglyceride and CRP levels were examined after Log_10_-transformation because they were highly skewed. *P*-values were determined by ANOVA, except * by χ^2^-test.

Multiple logistic analyses (Table 2) showed that, compared with the highest amylase quartile, lowest quartile was significantly associated with increased risk for MetS and diabetes even after adjustment for clinical confounders including eGFR.

**Table 2:** Associations between serum amylase quartiles with MetS and diabetes

	Model 1	Model 2	Model 3
Current amylase quartiles*	MetS		
Q1 (Lowest)	2.79 (2.04-3.82)	3.30 (2.38-4.57)	2.07 (1.39-3.07)
Q2	1.92 (1.38-2.66)	2.21 (1.58-3.09)	1.70 (1.14-2.54)
Q3	1.69 (1.22-2.35)	1.85 (1.32-2.58)	1.56 (1.05-2.31)
Q4 (Highest) reference	1	1	1
*P *for trend	0.02	0.02	0.03
	Diabetes		
Q1 (Lowest)	1.97 (1.32-2.93)	2.73 (1.79-4.18)	2.76 (1.49-5.11)
Q2	1.23 (0.79-1.89)	1.57 (1.00-2.46)	1.95 (1.03-3.68)
Q3	0.93 (0.59-1.47)	1.07 (0.67-1.70)	1.16 (0.59-2.29)
Q4 (Highest) reference	1	1	1
*P *for trend	0.13	0.08	0.03
Prior amylase quartiles (n = 571) **	MetS		
Q1 (Lowest)	4.21 (1.81-9.76)	4.82 (2.01-11.5)	5.88 (2.05-16.8)
Q2	3.37 (1.47-7.72)	3.78 (1.61-8.85)	3.97 (1.45-10.8)
Q3	1.22 (0.47-3.19)	1.34 (0.51-3.53)	1.68 (0.54-5.19)
Q4 (Highest) reference	1	1	1
*P *for trend	0.04	0.04	0.02
	Diabetes		
Q1 (Lowest)	3.70 (0.73-18.7)	3.74 (0.68-20.6)	3.26 (0.45-23.4)
Q2	0.92 (0.13-6.59)	0.91 (0.12-6.89)	0.60 (0.06-5.56)
Q3	1.46 (0.24-8.86)	1.65 (0.26-10.5)	1.78 (0.23-14.1)
Q4 (Highest) reference	1	1	1
*P *for trend	0.25	0.27	0.38

Model 1: unadjusted; Model 2: adjusted for age, sex, and current smoking (versus non-smokers); Model 3: Model 2 plus adjustment for infrequent exercise (versus frequent exercise), daily alcohol consumption (versus infrequent/no alcohol consumption), BMI, CRP and eGFR. *Current quartiles are identical to those in Table 1. The numbers of subjects in the previous amylase quartiles are 144, 149, 157, and 121 for Q1-Q4, respectively. **The ranges of values for the prior serum amylase quartiles are 30-55, 56-69, 70-85 and 86-198 IU/l for Q1-Q4, respectively.

### Retrospective analysis

**Figure 3:**
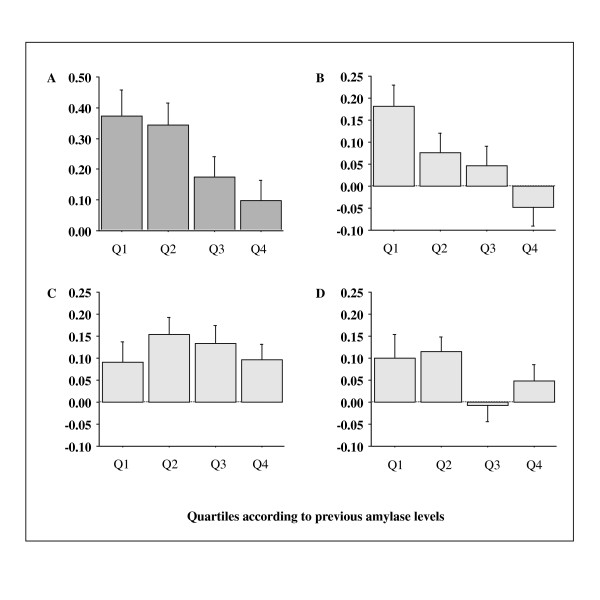
**Changes over 5 years for NAMC-BMI and its components**. A: NAMC-BMI, B: Proportion of elevated blood pressure, C: Proportion of high FPG, D: Proportion of dyslipidemia. The numbers of subjects in Q1, Q2, Q3 and Q4 are 144, 149, 157, and 121, respectively. NAMC-BMI and proportions of elevated blood pressure decreased significantly with increasing amylase quartile recorded 5 years ago (*P *= 0.02 and *P *= 0.006, ANOVA, respectively). No significant association was observed between prior quartiles of serum amylase and dyslipidemia and high FPG. NAMC: number of ATP-III-MetS components.

**Figure 4:**
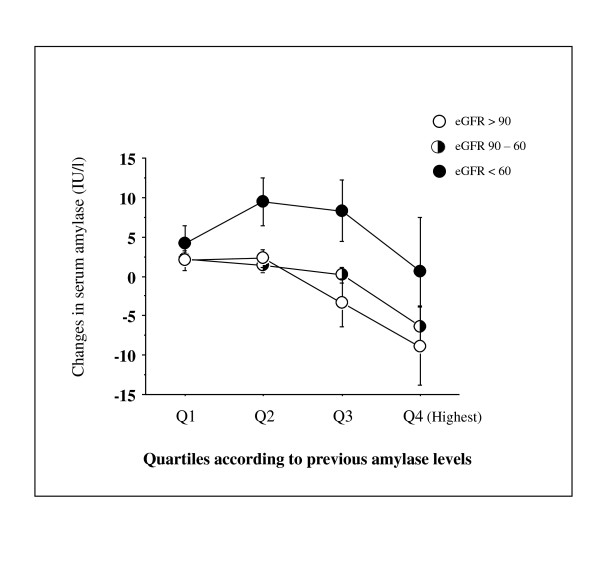
**Changes in serum amylase over 5 years**. The numbers of subjects in Q1, Q2, Q3 and Q4 [further divided according to current kidney functions as eGFR (ml/min/1.73 m^2^)] are 144 (25, 105, 14), 149 (16, 120, 13), 157 (17, 116, 24), and 121 (9, 86, 26), respectively. Current serum amylase increased significantly with decreasing amylase quartile recorded 5 years ago and decreasing current eGFR (*P *= 0.004 and *P *= 0.02, ANOVA, respectively). Values are means ± SE.

We next repeated the analysis in subjects who underwent the checkup 5 years ago (n = 571). In this analysis, BMI ≥ 25 kg/m^2 ^was used for the determination of current and previous MetS instead of elevated waist circumference. As shown in Table 2, compared with the highest amylase quartile at the previous checkup, the previous lowest amylase quartile was significantly associated with current MetS, but not current diabetes, which remained after adjustment for confounding factors recorded at the previous checkup. Figure 3 shows the changes over time for NAMC-BMI and its components according to previous amylase quartiles. Subjects with lower amylase levels 5 years ago showed significantly greater increases in NAMC-BMI (*P *= 0.02, Figure 3A) and proportions of elevated blood pressure (*P *= 0.006, Figure 3B). Figure 4 shows the changes over time for serum amylase according to previous amylase quartiles and three groups classified according to current eGFR. Overall a significant inverse association between changes in serum amylase and increasing previous amylase levels was observed (*P *< 0.0001). Although the serum amylase level in lowest quartile 5 years ago increased slightly at the recent checkup by ~5 IU/l, it was not affected by kidney dysfunction (eGFR < 60 ml/min/1.73 m^2^), compared with those in other quartiles.

## Discussion

Although the pathogenic features of reduced amylase secretion and the underlying mechanisms linking islets and acinar cells have been extensively examined in animal and cellular studies [14-19], clinical evidence supporting such exocrine-endocrine relationships has not been established in large human studies. Our results in this epidemiological study indicate that serum amylase levels are inversely associated with most cardiometabolic risk factors, especially those associated to obesity. Furthermore, the serum amylase level decreased with worsening of metabolic abnormalities and these declines were independent of smoking, a strong factor for increasing insulin resistance [25,26]. Meanwhile, the pattern of changes in plasma glucose levels during the OGTTs suggest that subjects with low serum amylase have lower glucose tolerance. Significant associations of current and previous low serum amylase with current MetS and diabetes (current low serum amylase only) were observed and remained after adjustment for confounding factors. Additionally, the severity of metabolic abnormalities worsened over 5 years in subjects with low serum amylase at the previous checkup. Accordingly, our results suggest that low serum amylase levels may reflect metabolic abnormalities and abnormal glucose metabolism, both of which are associated with impaired insulin action due to insulin resistance and/or inadequate insulin secretion.

In terms of cardiometabolic risk factors, only kidney dysfunction contributes to elevations in serum amylase levels because the kidney plays the main role in eliminating circulating amylase [2-6]. Nevertheless, the fluctuation in serum amylase levels in subjects in the lowest quartile 5 years ago was not affected by kidney dysfunction (Figure 4), suggesting that low serum amylase may be a stable marker for cardiometabolic disorders related to impaired insulin action. One reason for the decrease over 5 years in the highest quartile 5 years ago is that insulin sensitivity decreased or kidney function improved before the current checkup. The most plausible explanation for the significant increase in proportions of elevated blood pressure is that resultant hyperinsulinemia may affect Na+ reabsorption by the kidney and circulating volume retention, leading to increased blood pressures. In contrast, dyslipidemia and abnormal FPG in subjects in the lowest quartile were already evident and did not deteriorate thereafter, as compared with those in other quartiles. Furthermore, the small sample size in cohort data may interfere with the outcomes. Noteworthy, the finding that prevalence of history of stroke was increased in subjects with higher amylase (Table 1) may be contradicting. However, this might be consistent with the recent report that reduced eGFR, which was observed with increasing serum amylase in this study, was associated with increased intima-media thickness of the carotid artery in patients with type 2 diabetes [27]. Furthermore, it is unclear why the prevalence of treatment for hypercholesterolemia mostly with statins was higher in subjects with higher amylase levels (Table 1). Therefore, large cross-sectional and prospective studies will be needed for the exploration of relationship between low serum amylase and metabolic abnormalities as well as the treatment with medications.

Regarding the cause-effect relationship, low serum amylase levels were believed to be due to deficient insulin activity [10-13], which is consistent with early findings that insulin and ciglitazone (this drug was never approved for clinical use), a peroxisome proliferator-activated receptor-γ agonist respectively increased and restored the secretion of amylase from pancreases isolated from rats [15,16]. In contrast, the results of our retrospective longitudinal analysis suggest that low serum amylase levels preceded the overt metabolic abnormalities. Alternatively, as proposed by Dandona et al. [11], a common pathological process exists in the pancreas that simultaneously affects the endocrine and exocrine components of the pancreas. Consistent with this notion, Hayden et al. [28] suggested that a continuous interstitial matrix connection between endocrine and exocrine is lost in animal models and humans with type 2 diabetes, resulting in a dysfunctional insulino-acinar-ductal-incretin gut hormone axis. Besides these putative mechanisms, there are multiple defects in insulin secretion and signaling in type 2 diabetes [24,28], which might be associated with the low amylase secretion from the pancreas.

Several limitations of this study should be mentioned. First, the blood insulin levels were not measured. However, previous studies have shown positive correlations between amylase secretion and circulating C-peptide or 24-hr urinary C-peptide excretion in diabetic patients, principally in type 1 diabetes [10-12], suggesting that low circulating amylase may reflect low insulin secretion. Nevertheless, obese people with MetS and type 2 diabetes tend to show hyperinsulinemia to compensate for insulin resistance. Therefore, whether low serum amylase levels are truly associated with hypoinsulinemia or hyperinsulinemia is unclear. Measurement of insulin levels would help confirm the associations observed in this study, including the weak association between HbA1c and serum amylase. Second, although it has been reported that pancreatic serum amylase secretion was decreased in the presence of hyperglycemia [2], the actual level of pancreatic amylase relative to salivary amylase in subjects with low 'total serum amylase' is unknown and should be determined.

In conclusion, our study demonstrated that serum amylase levels may be inversely related with many cardiometabolic risk factors. Furthermore, low serum amylase levels may be unchangeable even in the presence of kidney dysfunction and are independently associated with MetS and diabetes, suggesting a possible exocrine-endocrine relationship in various clinical conditions. The causality, underlying mechanisms, and whether low serum amylase is a valuable marker for these states remain to be elucidated.

## Competing interests

The authors declare that they have no competing interests.

## Authors' contributions

KN, HF and HM designed the study; KN, TN, and HF collected and analyzed the data; KN, TM and MK researched and evaluated the literature; and KN wrote the first draft of the manuscript. All authors reviewed and edited the manuscript, and approved the final version of the manuscript.
